# The Pressurised Leaky Funnel: A Systems Framework for Recruitment, Selection and Retention in UK Psychiatry

**DOI:** 10.1192/bjo.2026.11303

**Published:** 2026-06-30

**Authors:** Jun Jie Lim, Adrian Lloyd, Ananta Dave, Hugh Alberti, Gill Vance

**Affiliations:** 1 School of Medicine, Newcastle University, Newcastle upon Tyne, United Kingdom; 2 Cumbria, Northumberland, Tyne and Wear NHS Foundation Trust, Newcastle upon Tyne, United Kingdom; 3 Health Education England, Newcastle upon Tyne, United Kingdom; 4 NHS Black Country Integrated Care Board, Wolverhampton, United Kingdom

## Abstract

**Aims:**

Recent increases in competition ratios for UK psychiatry training suggest recruitment success, yet consultant vacancies and attrition remain high. Thisfeatureaims to explore why recruitment gains have failed to translate into workforce sustainability and to propose a systems-based framework for understanding recruitment, selection, and retention as an integrated process.

**Methods:**

We synthesised UK psychiatry workforce data, national recruitment statistics, and medical education literature to examine progression and attrition across the training pathway. Drawing on theories from workforce planning, medical education, and organisational psychology, we developed a conceptual model integrating recruitment pressures with downstream attrition.

**Results:**

We identify a recruitment–retention paradox in which rising applicant volumes coexist with persistent workforce shortages. Competition ratios and fill rates obscure cumulative attrition across training and career transitions, particularly at the core-to-higher training interface. We introduce the Pressurised Leaky Funnel, a model illustrating how upstream recruitment pressures amplify applicant volume while downstream structural leaks erode workforce capacity over time.

**Conclusion:**

Workforce sustainability in psychiatry cannot be achieved through recruitment-led strategies alone. Reframing recruitment, selection, and retention as interacting components of a single system highlights the need for better alignment between selection processes, training environments, and long-term role sustainability. The Pressurised Leaky Funnel offers a framework to guide more coherent workforce planning, evaluation, and reform in UK psychiatry.

